# C1QTNF6 regulated by miR‐29a-3p promotes proliferation and migration in stage I lung adenocarcinoma

**DOI:** 10.1186/s12890-022-02055-2

**Published:** 2022-07-25

**Authors:** Guofu Lin, Lanlan Lin, Hai Lin, Yingxuan Xu, Wenhan Chen, Yifei Liu, Jingyang Wu, Shaohua Chen, Qinhui Lin, Yiming Zeng, Yuan Xu

**Affiliations:** 1 Department of Pulmonary and Critical Care Medicine, The Second Affiliated Hospital of Fujian Medical University, Quanzhou, 362000 Fujian Province China; 2 Respiratory Medicine Center of Fujian Province, Quanzhou, 362000 Fujian Province China; 3 The Second Clinical College, Fujian Medical University, Fuzhou, 350004 Fujian Province China; 4 Clinical Center for Molecular Diagnosis and Therapy, The Second Affiliated Hospital of Fujian Medical University, Quanzhou, 362000 Fujian Province China; 5 Department of Thoracic Surgery, The Second Affiliated Hospital of Fujian Medical University, Quanzhou, 362000 Fujian Province China; 6 Department of Pathology, The Second Affiliated Hospital of Fujian Medical University, Quanzhou, 362000 Fujian Province China

**Keywords:** C1QTNF6, Lung adenocarcinoma, miR‐29a-3p, Cytokine-cytokine receptor interaction pathway

## Abstract

**Objective:**

C1QTNF6 has been implicated as an essential component in multiple cellular and molecular preliminary event, including inflammation, glucose metabolism, endothelial cell modulation and carcinogenesis. However, the biological process and potential mechanism of C1QTNF6 in lung adenocarcinoma (LUAD) are indefinite and remain to be elucidated. Therefore, we investigated the interaction among the traits of C1QTNF6 and LUAD pathologic process.

**Methods:**

RT-qPCR and western blot were conducted to determine the expression levels of C1QTNF6. RNA interference and overexpression of C1QTNF6 were constructed to identify the biological function of C1QTNF6 in cellular proliferative, migratory and invasive potentials in vitro. Dual-luciferase reporter assay was applied to identify the possible interaction between C1QTNF6 and miR‐29a-3p. Moreover, RNA sequencing analysis of C1QTNF6 knockdown was performed to identify the potential regulatory pathways.

**Results:**

C1QTNF6 was upregulated in stage I LUAD tissues compared with adjacent non-cancerous tissues. Concurrently, C1QTNF6 knockdown could remarkably inhibit cell proliferation, migratory and invasive abilities, while overexpression of C1QTNF6 presented opposite results. Additionally, miR‐29a-3p may serve as an upstream regulator of C1QTNF6 and reduce the expression of C1QTNF6. Subsequent experiments showed that miR‐29a-3p could decrease the cell mobility and proliferation positive cell rates, as well as reduce the migratory and invasive possibilities in LUAD cells via downregulating C1QTNF6. Moreover, RNA sequencing analysis demonstrated that the cytokine-cytokine receptor interaction pathway may participate in the process of C1QTNF6 regulating tumor progression.

**Conclusion:**

Our study first demonstrated that downregulation of C1QTNF6 could inhibit tumorigenesis and progression in LUAD cells negatively regulated by miR‐29a-3p. These consequences could reinforce our awareness and understanding of the underlying mechanism and provide a promising therapeutic target for LUAD.

**Supplementary Information:**

The online version contains supplementary material available at 10.1186/s12890-022-02055-2.

## Introduction

Lung cancer remains the leading cause of cancer related death worldwide [1]. Non-small cell lung cancer (NSCLC) is the predominant subtype, representing about 85% of diagnosed lung cancer cases [2]. Traditional surgery resection is the optimal therapeutic option for patients with early-stage NSCLC. Heterogeneity of pathologic stage is one of the major causes of recurrence and diverse prognosis of LUAD patients. The rate of postoperative recurrence for stage I NSCLC remains unfavorable [3]. Previous reports showed that 30%-35% stage I NSCLC patients treated with surgery may progress to local recurrence or distant metastasis within five years [4]. The 5-year survival rate is 83.9% in stage IA and 66.3% in stage IB NSCLC [5]. Lung adenocarcinoma (LUAD) is the most common histologic type of NSCLC with morbidity and mortality persistently elevated over past decades [6]. Therefore, identification of the potential pathogenic genes and underlying mechanisms to predict recurrence and progression of early-stage LUAD is of great significance.

Previous studies have demonstrated that C1q/tumor necrosis factor-related proteins (C1QTNFs, also called CTRPs) may modulate various biological events, including cellular development, differentiation, metabolism, proliferation and apoptosis [7–10]. C1QTNF6, a member of C1QTNFs family, is primarily expressed in adipose tissues, liver, lung and so on [11–13], which is mainly involved in inflammation, glucose metabolism, endothelial cell modulation and carcinogenesis and so on [13–16]. Amongst, the biological role of C1QTNF6 in malignant tumors has become a hot research topic in recent years. It has been reported that C1QTNF6 was upregulated in hepatocellular carcinoma (HCC) and inhibition of C1QTNF6 could prevent survival, migration and promote apoptosis in HCC cells by inactivation of the AKT signaling pathway. Similarly, C1QTNF6 mRNA and protein expressions were found to be remarkably high in bladder cancer (BC) and higher C1QTNF6 expression may predict as an adverse prognostic marker for BC patients [17]. Although emerging evidence has indicated C1QTNF6 modulated cellular apoptosis in lung cancer [18], the biological process and underlying mechanism of C1QTNF6 in LUAD still remain unknown.

MicroRNAs (miRNAs), as a group of highly conserved non-coding small RNAs, can modulate gene post-transcriptional regulation by targeting messenger RNAs (mRNAs) [19]. miRNAs have been identified as essential regulators in cellular processes, including cell proliferation, differentiation and apoptosis [20–22]. Dysregulation and dysfunction of miRNAs play a critical role in tumor pathogenesis [23–26]. MiR‐29a-3p, a newly discovered tumor-related intergenic miRNA, has been predicted as a potential key regulator of C1QTNF6 by bioinformatics analysis. Accumulating studies have reported that miR‐29a-3p may promote several tumors pathogenesis and progression, such as gastric cancer [27], liver cancer [28], prostate cancer and so on [29]. While the detailed function of miR‐29a-3p in LUAD tumorigenesis has not been well explored.

Currently, we aimed to investigate the potential function of C1QTNF6 and miR‐29a-3p on LUAD cell lines, and to reveal the underlying relationship between C1QTNF6 and miR‐29a-3p. Additionally, we further investigated the underlying downstream pathway of C1QTNF6 by RNA sequencing. These findings might provide an available theoretical basis to enhance the biological targeted remedy of LUAD.

## Materials and methods

### Ethics

This study was approved by The Second Affiliated Hospital of Fujian Medical University Academic Ethics Committee (2020-206). All patients have ever provided their written, informed consent. Current study was performed in accordance with the ethical principles of the Declaration of Helsinki.

### Tissue samples

Paired LUAD tissues and adjacent non-cancerous samples were collected from The Second Affiliated Hospital of Fujian Medical University with the informed consent between 2017 and 2020. These patients had been histologically and pathologically diagnosed as stage I LUAD. All tissues were obtained from surgical resection at the Department of Thoracic Surgery. None of the patients received any pre-operative chemotherapy or radiotherapy prior to tissue sampling. All collected pulmonary tissue samples were permanently stored in a − 80 °C freezer until use.

### Cell culture

Five human LUAD cell lines (H1975, H1299, A549, SPCA-1 and H460), BEAS-2B and HEK-293T cells were purchased from the American Type Culture Collection (ATCC, Manassas, VA, USA). The cell lines were supplemented with RPMI-1640 or DMEM medium (Gibco, USA) containing 10% fetal bovine serum (FBS; Gibco, USA), 100 U/ml penicillin and 100 mg/ml streptomycin and further cultured at 37 °C and 5% CO_2_ atmosphere.

### Quantitative reverse transcription polymerase chain reaction (RT-qPCR)

Total RNA was extracted and purified from tissues or cells using Trizol reagent (Invitrogen, USA). 1000ng of RNA were subjected to RT-qPCR system using the Takara RT-qPCR kit (Takara, Japan). Reactions were carried out in a total of 20 μL solution. To determine the level of mature miRNA, we used a stem-loop RT primer. The RT-qPCR was conducted with three independent replicates and the relative gene expression level was analyzed using the comparative 2^−ΔΔCT^ method [30]. All the primers applied in this study were listed in Additional file 1: Table S1.

### Immunohistochemistry assay (IHC)

Collected tissues were fixed in 4% paraformaldehyde overnight at 4 ℃ and embedded in paraffin for immunohistochemical analysis. Paraffin-embedded pulmonary sections were deparaffinized and rehydrated. The slides were boiled in citrate buffer (pH 6.0) for 3 min for antigen retrieval. Endogenous peroxidase was then blocked using 3% hydrogen peroxide for 20 min at room temperature. After washing with PBS (pH 7.4) for 3 times, the sections were blocked with goat serum for 1 h followed by incubation with primary antibodies for 2 h at room temperature. Next, the HRP-conjugated goat anti-rabbit antibody and DAB (OriGene, Beijing, China) were applied. Finally, the slides were counterstained with hematoxylin and mounted. The following antibody was used: C1QTNF6 antibody (1:400, Bioss, China).

### Western blot analysis

Western blot analysis was performed as previously described [31]. LUAD tissues and cell lines were lysed in RIPA buffer containing protease/phosphatase inhibitor (Beyotime, China). The samples were grinded with a mechanical homogenizer and centrifuged for 15 min, 12,000 rpm at 4 °C. Protein samples were further electrophoresed on corresponding concentration of SDS-PAGE gels and transferred to PVDF membranes (Millipore, Eschborn, Germany). The membrane was blocked for 2 h at room temperature with 5% milk in Tris-buffered saline (TBS) with 0.1% Tween 20 (Biofroxx, Germany) before incubating with primary antibody overnight at 4 °C. Subsequently, an appropriate HRP-conjugated secondary antibody was incubated for 1 h at room temperature. Western blot was visualized with chemiluminescence reagents (Biosharp, China).

### Cell proliferation assay

Cell Counting Kit-8 (CCK-8, Beyotime, China) assay and EdU (Beyotime, Shanghai, China) incorporation assay were performed to evaluate cell proliferation as described in previous study [32].

Additionally, cell cycle assay was also conducted. The detailed procedures were as follows. Firstly, LUAD cells were digested by EDTA-free Trypsin and collected by centrifugation for 5 min at 1000 rpm. And then, collected cells were washed once with PBS. For cell cycle analysis, cells were fixed in 75% ethanol overnight at 4 °C and added 100 μL RNaseA for 30 min at 37 °C, and incubated with 200 µL propidium iodide (PI) for 30 min at 4 °C in the dark. Cell cycle distribution was measured by flow cytometry (BD Biosciences, San Diego, CA, USA). A total of 1.0 × 10^4^ events were acquired for analysis using the FlowJo V10 software.

### Cell migration and invasion assays

Cell migration and invasion assays were conducted using Boyden chambers (8 μm pore size; BD Falcon, 353097) with or without Matrigel (Corning Matrigel® Matrix, Corning, NY, USA). For cell migration assays, cells were resuspended in 200 μL serum-free medium (a total of 5.0 × 10^4^ cells) and added into the upper chamber. Medium with 20% FBS was added to the lower chamber. After 48 h incubation, non-migrated cells were removed from the upper surface carefully using cotton swabs, and the migrated cells were fixed with methanol, stained with 0.1% crystal violet solution for 20 min, photographed under microscope and quantified. Similarly, Transwell inserts were pre-coated with Matrigel on the upper layers were used to determine the invasive potentials.

Additionally, scratch assay was also performed to determine cell migration. 4.0 × 10^5^ A549 and H1975 cells were seeded in 6-well plates to reach 100% confluence within 24 h. Subsequently, cells were scraped in a straight line using a 200 μL pipette tip, washed using PBS and then replaced with serum-free medium. Photographs were captured under microscope (Olympus, Tokyo, Japan) at 0 h and 48 h respectively. The scratch healing rate was determined by comparing the size of scratch area using Image J software. *P *< 0.05 was considered as statistically different.

### Cell transfection

miR-29a-3p mimics, miR-29a-3p inhibitor, and corresponding control, plasmid of C1QTNF6 overexpression and small interfering RNA targeting C1QTNF6 (si- C1QTNF6) were synthesized by Hanheng Biotechnology Co., Ltd. (Shanghai, China). Cells were seeded one day before transfection with plasmid DNA, siRNA, or miRNA inhibitor/mimics using Lipofectamine 3000 (Invitrogen, Carlsbad, CA, USA). After transfection for 24 h, cells were harvested to identify the knockdown efficiency by RT-qPCR. The sequences were listed in Additional file 1: Table S1.

### Dual-luciferase reporter assay

psi-CHECK2 vector (Promega, USA) with the wild type (WT) C1QTNF6 sequence (C1QTNF6-WT) and mutant C1QTNF6 sequence (C1QTNF6-MUT) (within predicted miR-29a-3p binding sites) were constructed to verify the binding sites between C1QTNF6 and miR-29a-3p. Subsequently, the miR-NC or miR-29a-3p was co-transfected into 293T cells with luciferase reporter vectors for the execution of the dual-luciferase reporter assay, respectively. Luciferase reporter activity was determined using dual-luciferase reporter assay kit (Promega) after transfecting 48 h with firefly luciferase values normalized to renilla luciferase values.

### Transcript data analysis

Total RNA of 10 paired stage I LUAD tissues, corresponding normal tissues and lung adenocarcinoma cell lines was extracted using RNeasy Mini Kit (Qiagen, Germany) according to the manufacturer’s protocol. Then, rRNA was removed from total RNA to obtain the maximum residual ncRNA. After fragment of rRNA-depleted RNA, the cDNA library was performed using the TruSeq RNA sample Prep Kit (Illumina, USA). mRNA sequencing libraries were prepared using VAHTS total RNA-seq Library Prep kit for Illumina (Vazyme NR603, China) following the manufacturer’s protocol. After sequencing was completed, the reads files (fastq) were mapped to the Hg19 reference using STAR, and gene expression was determined using RSEM. Differential expression analysis for mRNA was performed using DESeq2 R package (https://bioconductor.org/packages/release/bioc/html/DESeq2.html). Differentially expressed genes (DEGs) were obtained by comparing gene expressions between two groups using DESeq2 (v1.10.1). Corrected *p* value of < 0.05 and |Log2(fold change)| (|log2FC|) ≥ 1 were considered statistically significant. Heat maps were generated by R package with hierarchical clustering algorithm.

### Gene ontology and KEGG pathway enrichment analysis

The DEGs were annotated with gene ontology (GO) terms to explore putative functions. GO enrichment analysis was performed using the R package TopGO [33]. The statistical significance of GO terms was analyzed using Fisher’s exact test. Kyoto Encyclopedia of Genes and Genomes (KEGG) (https://www.kegg.jp/kegg/kegg1.html) [34] pathway was performed using KOBAS 3.0 (http://kobas.cbi.pku.edu.cn/) with an enrichment *p* value < 0.05.

### Statistical analysis

Statistical analysis was performed using the Student *t* test for parametric data and the Mann-Whitney test for non-parametric data. All data were expressed as mean ± SD. *P *< 0.05 was considered as significant. The experiments were repeated independently three times.

## Results

### C1QTNF6 was upregulated in LUAD tissues and cells

**Fig. 1:**
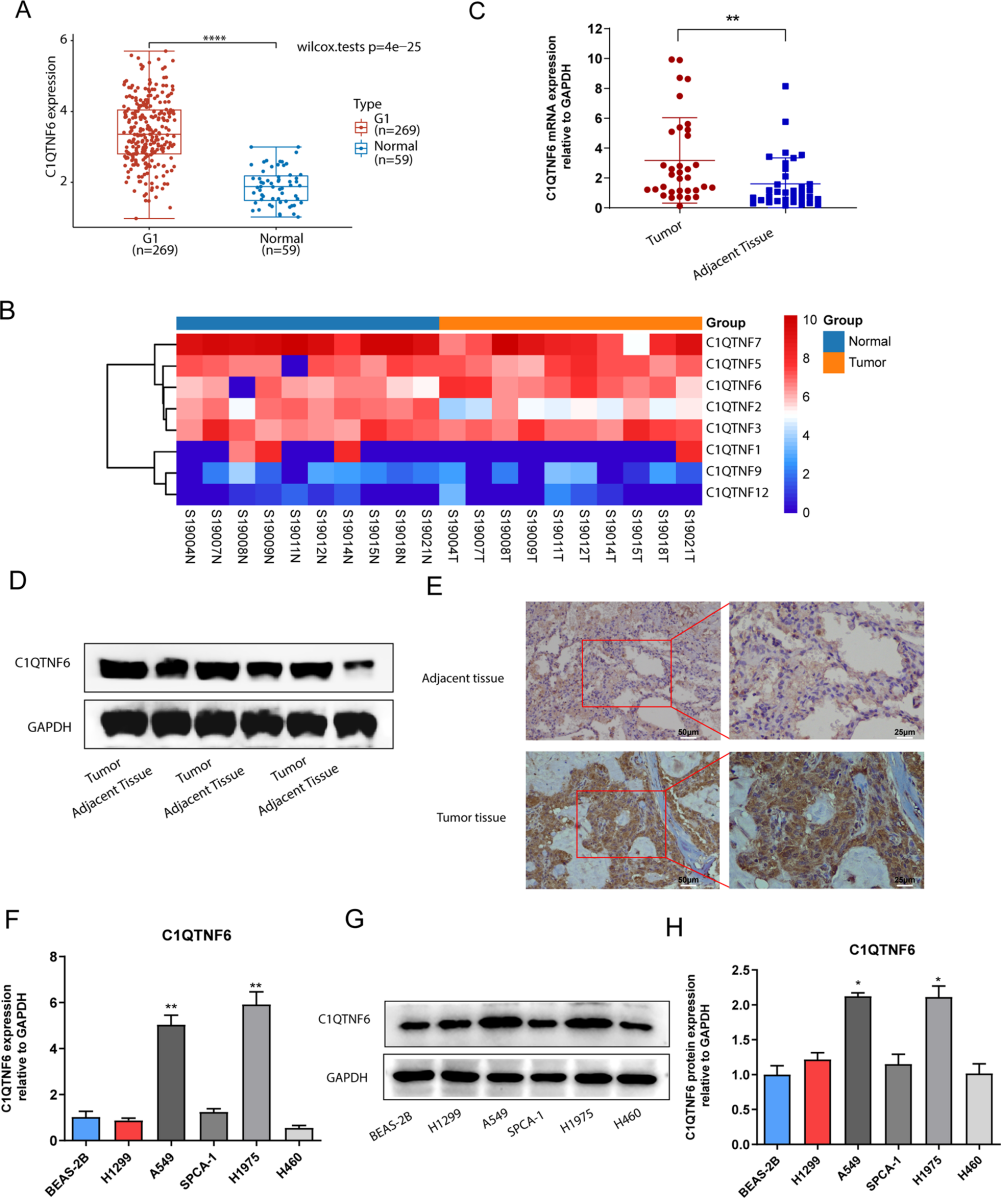
C1QTNF6 was upregulated in stage I LUAD tissues. **A** Boxplots of C1QTNF6 mRNA expression between normal (n = 59) and stage I LUAD tissues (n = 269) in the TCGA dataset. **B** Heat maps of C1QTNFs expression profiles in stage I LUAD and adjacent tissues based on sequencing results. **C** The expression of C1QTNF6 in stage I LUAD and paired adjacent tissues was detected with RT-qPCR (n = 34). **D**, **E** The protein expression of C1QTNF6 in LUAD and adjacent non-tumor tissues was evaluated using western blot (n = 3) and IHC staining (n = 9). **F** C1QTNF6 mRNA levels in different LUAD cell lines. **G**, **H** Expression levels of C1QTNF6 protein in different LUAD cell lines by western blot. G1, stage I LUAD. (*p < 0.05, **p < 0.01, ****p < 0.0001)

Based on public data deposited in The Cancer Genome Atlas (TCGA; http://tcga-data.nci.nih.gov/tcga/), we found that C1QTNF6 mRNA expression was significantly upregulated in stage I LUAD compared with adjacent tissues (Fig. 1A). Furthermore, we performed high-throughput sequencing in 10 pairs of stage I LUAD and adjacent non-cancerous lung tissues. The heat map clearly showed C1QTNFs-related cluster (Fig. 1B), which exhibited a significant high C1QTNF6 expression compared with matched adjacent tissue (*p *< 0.001). And then, C1QTNF6 mRNA expression in 34 paired stage I LUAD tissues and adjacent non-tumorous pulmonary tissues exhibited that C1QTNF6 was significantly higher in stage I LUAD tissues (Fig. 1C), which was consistent with the previous results of TCGA and RNA-sequencing.

Moreover, C1QTNF6 protein expression was detected by western blot and immunohistochemical assay. The results demonstrated that C1QTNF6 protein was significantly overexpressed in the lung tissues of stage I LUAD patients (n = 3) compared with adjacent non-cancerous tissues (Fig. 1D). Immunohistochemical data confirmed the elevated expression of C1QTNF6 in stage I LUAD tissues (Fig. 1E).

Additionally, we also identified the expression of C1QTNF6 in LUAD cell lines and normal lung epithelial cell lines and observed that the mRNA and protein levels of C1QTNF6 were upregulated in LUAD cells lines (especially in A549 and H1975) than in lung epithelial cell line BEAS-2B (Fig. 1F–H).

### C1QTNF6 knockdown inhibited cell proliferation, migration and invasion in LUAD cells

**Fig. 2:**
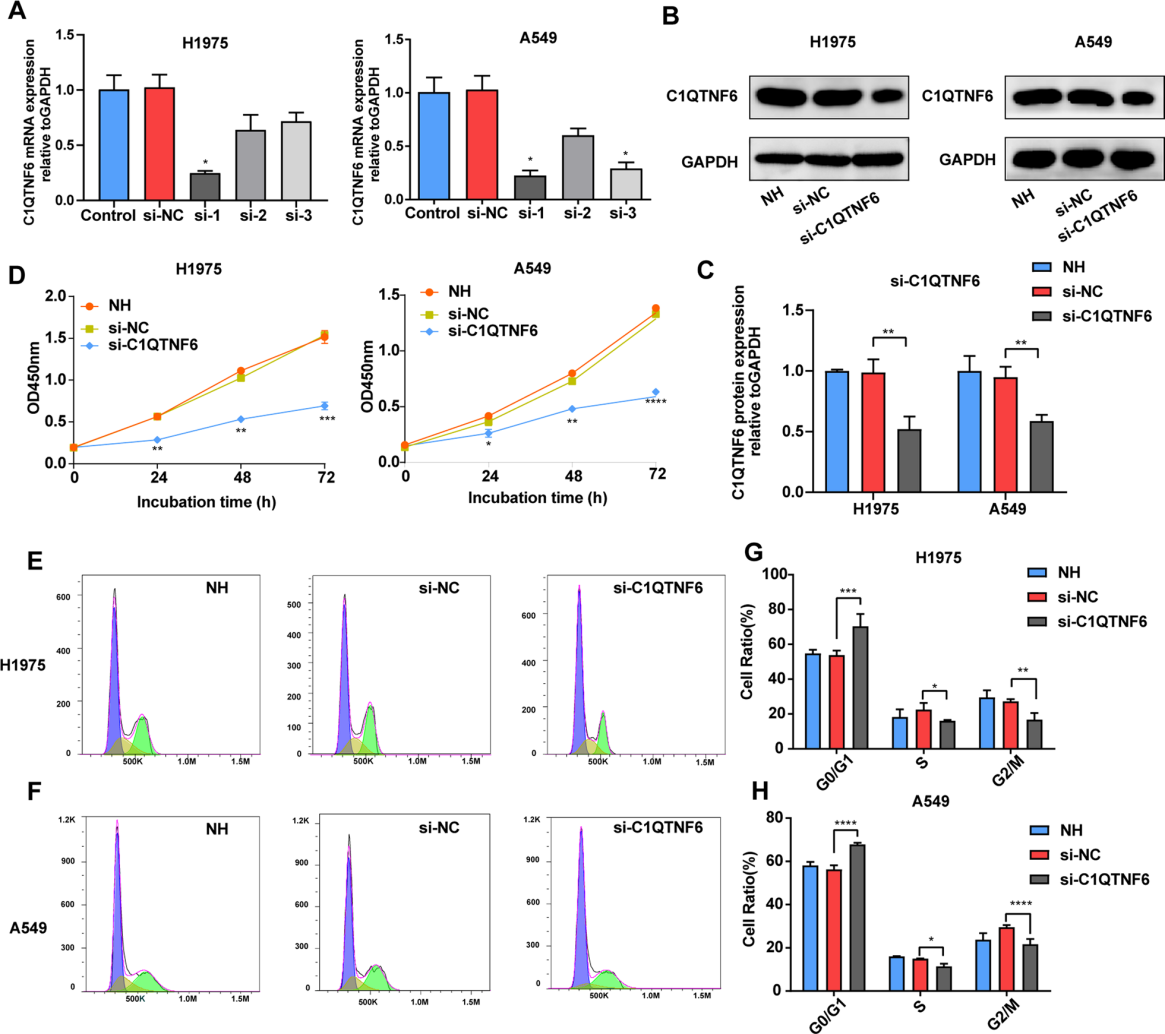
Knockdown of C1QTNF6 inhibited cell proliferation. **A** Knockdown efficacy in H1975 and A549 cells was identified by RT-qPCR after siRNA transfection. **B**, **C** Western blot analysis of C1QTNF6 expression was performed in H1975 and A549 cells transfected with si-C1QTNF6. **D** Growth curves in C1QTNF6 knockdown and control cells were measure by CCK-8. **E**–**H** The cell cycles of H1975 and A549 cells after transfection with si-C1QTNF6 and si-NC were analyzed using flow cytometry. NC, negative control. n = 3. (Data were presented as the mean ± SD of three independent experiments. *p < 0.05, **p < 0.01, ***p < 0.001, ****p < 0.0001)

To further explore the role of C1QTNF6 in LUAD cell lines, si-NC, si-C1QTNF6 (si-1, si-2 and si-3) were transfected into H1975 and A549 cells separately. The result of RT-qPCR indicated that mRNA expression of C1QTNF6 markedly decreased after transfecting si-C1QTNF6 (especially in si-1) in LUAD cells (Fig. 2A). Furthermore, western blot also verified that C1QTNF6 decreased at the protein level after siRNA transfection in H1975 and A549 cell lines (Fig. 2B, C).

Next, we explored whether C1QTNF6 suppression could affect the proliferation of LUAD cells. Intriguingly, CCK-8 assay demonstrated that the proliferation ability of H1975 and A549 cells was remarkably restrained after C1QTNF6 knockdown (Fig. 2D). Additionally, flow cytometric analysis suggested silence of C1QTNF6 could significantly inhibit the process of cell cycle and particularly suppress it at the G2/M phase (Fig. 2E–H).

**Fig. 3:**
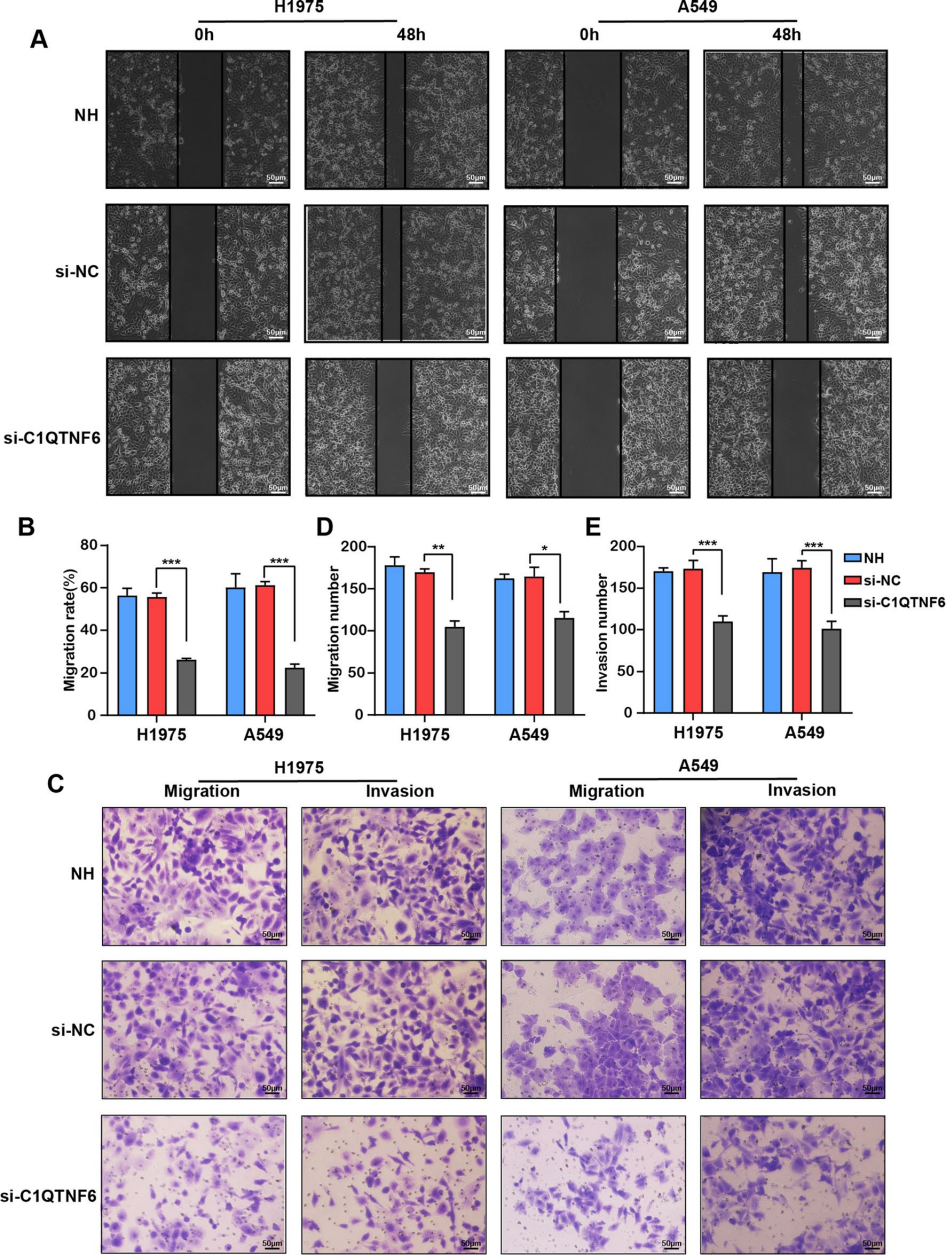
C1QTNF6 silencing suppressed migratory and invasive abilities in LUAD cells. **A**,** B** Scratch experimental results of H1975 and A549 cells after transfection with si-C1QTNF6, scale bar 50 µm. **C**–**E** Invasion and migration assays were performed with or without Matrigel after transfection with si-C1QTNF6 in H1975 and A549 cells, scale bar 50 µm. NC, negative control. n = 3. (Data were presented as the mean ± SD of three independent experiments. *p < 0.05, **p < 0.01, ***p < 0.001)

Migratory and invasive behaviors are typical property of malignant tumors. Therefore, cellular migration and invasion were further performed via wound healing and Transwell assays, respectively. It was found that C1QTNF6 silence for 48 h significantly decreased the migration rate in H1975 and A549 cells (Fig. 3A, B). Concomitantly, the results of Transwell assays suggested that knockdown of C1QTNF6 led to a significant reduction in the migration and invasion of H1975 and A549 cells (Fig. 3C–E).

### C1QTNF6 overexpression promoted cell growth and the migratory and invasive abilities in LUAD cells

In order to get insight into the function of C1QTNF6, we performed a gain-of-function approach to overexpress C1QTNF6 in H460 cells. The construction efficacy was verified by the elevated level of C1QTNF6 mRNA and protein expression. CCK-8 and cell cycle assays indicated that the proliferation ability of C1QTNF6 overexpression cells was significantly enhanced when compared with the control cells. Additionally, Transwell assay and wound healing assay demonstrated that overexpression of C1QTNF6 promoted the migratory and invasive abilities of H460 cells (Additional file 1: Fig. S1). Collectively, these data strongly suggested that C1QTNF6 exerted critical role in LUAD occurrence and progression.

### Identification of miR-29a-3p as potential upstream regulator of C1QTNF6

**Fig. 4:**
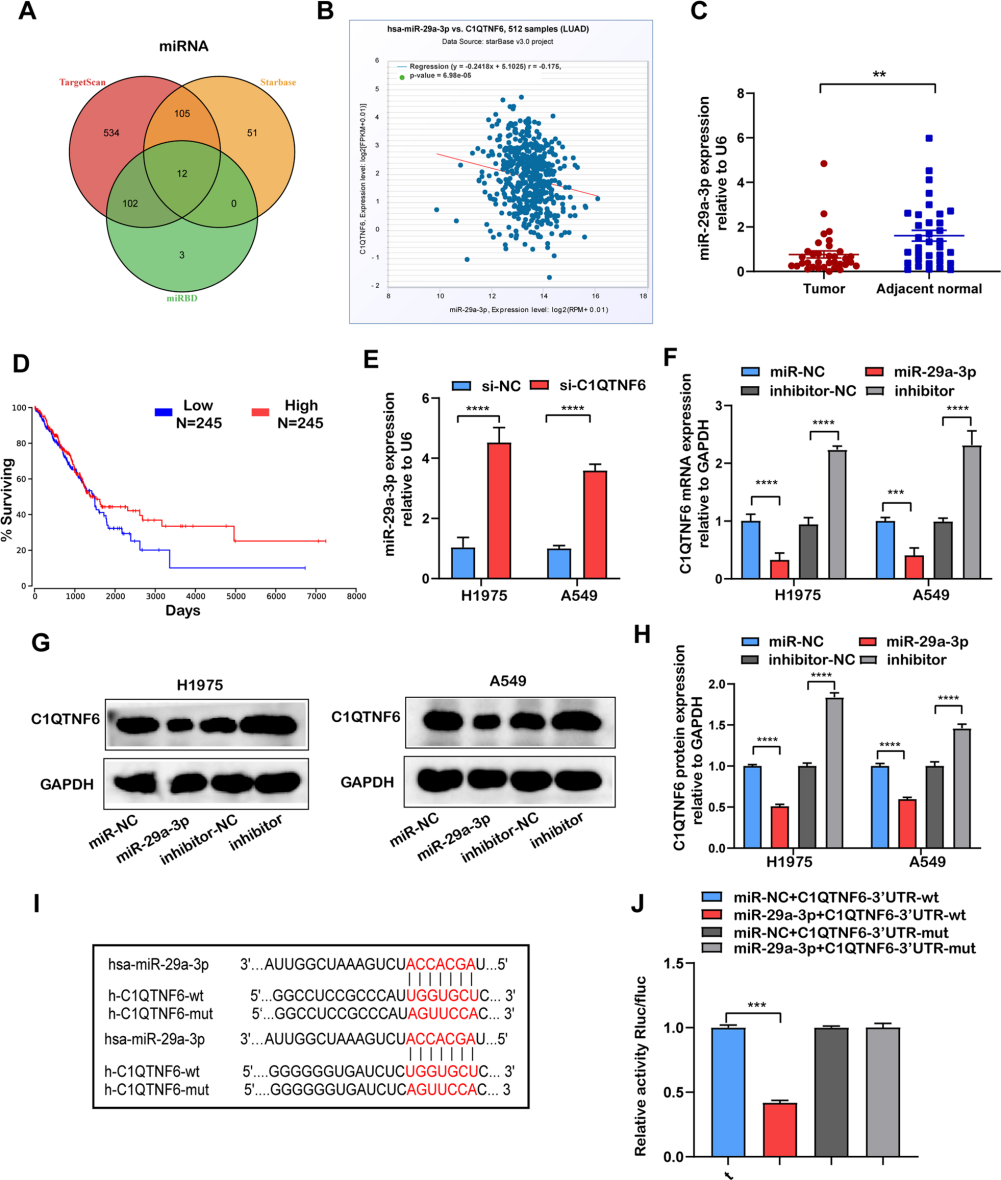
Relationship between miR‐29a-3p and C1QTNF6 was confirmed. **A** The upstream regulators of C1QTNF6 were discovered by a prediction website. **B** Correlations between expression of miR‐29a‐3p and C1QTNF6 in TCGA database. **C** The expression of miR‐29a‐3p in stage I LUAD and paired adjacent tissues was detected by RT-qPCR. **D** Kaplan‐Meier survival analysis of miR‐29a‐3p based on the TCGA LUAD data. **E** MiR-29a-3p expressions of H1975 and A549 cells after suppressing C1QTNF6 was evaluated using RT-qPCR. **F** C1QTNF6 mRNA expressions of H1975 and A549 cells after overexpression or knockdown of miR-29a-3p was evaluated using RT-qPCR. (**G-H**) C1QTNF6 protein expressions of H1975 and A549 cells after overexpression or knockdown of miR-29a-3p was evaluated using western blot. **I** Schematic representation of miR-29a-3p binding sites in the C1QTNF6 3'UTR. **J** The reporter activity was detected by dual-luciferase reporter system, luciferase reporter activity is normalized to that of renilla luciferase. miR‐29a-3p, miR‐29a-3p mimics; NC, negative control; wt: wide type; mut: mutated. n = 3. (Data were presented as the mean ± SD of three independent experiments. **p < 0.01, ***p < 0.001, ****p < 0.0001)

In order to identify biological molecule that might regulate C1QTNF6 in LUAD cells, miRDB, starBase and TargetScan were used to identify C1QTNF-related miRNAs. Venn diagram showed the number of overlapping miRNAs potentially targeting C1QTNF6 (Fig. 4A). Moreover, correlation analysis from starBase and RT-qPCR of clinical LUAD samples revealed that only miR-29a-3p was negatively correlated with C1QTNF6 and significantly downregulated in stage I LUAD tissues when compared with adjacent tissues (Fig. 4B, C). Additionally, low expression of miR-29a-3p predicted a worse prognosis in LUAD cases, which indicated that miR-29a-3p might play a potential role in progression of LUAD (Fig. 4D).

Next, we detected the miR-29a-3p expression after transfecting si-C1QTNF6 by RT-qPCR. The result showed that miR-29a-3p expression significantly increased in H1975 and A549 cell lines after suppressing C1QTNF6 when compared with control groups (Fig. 4E). Interestingly, the mRNA and protein expressions of C1QTNF6 also altered dramatically by transfecting miR-29a-3p or miR-29a-3p inhibitor (Fig. 4F–H). Furthermore, we performed a dual luciferase reporter assay. The dual-luciferase reporter assay demonstrated that the 3'UTR of C1QTNF6 bound to miR-29a-3p (Fig. 4I). Compared with the WT-C1QTNF6 + miR-NC group, the WT-C1QTNF6 + miR-29a-3p group significantly reduced relative luciferase activity (*p *< 0.05; Fig. 4J). These data indicated that C1QTNF6 was a target gene of miR-29a-3p in vitro.

### MiR-29a-3p inhibited LUAD cells growth and migration by targeting C1QTNF6

**Fig. 5:**
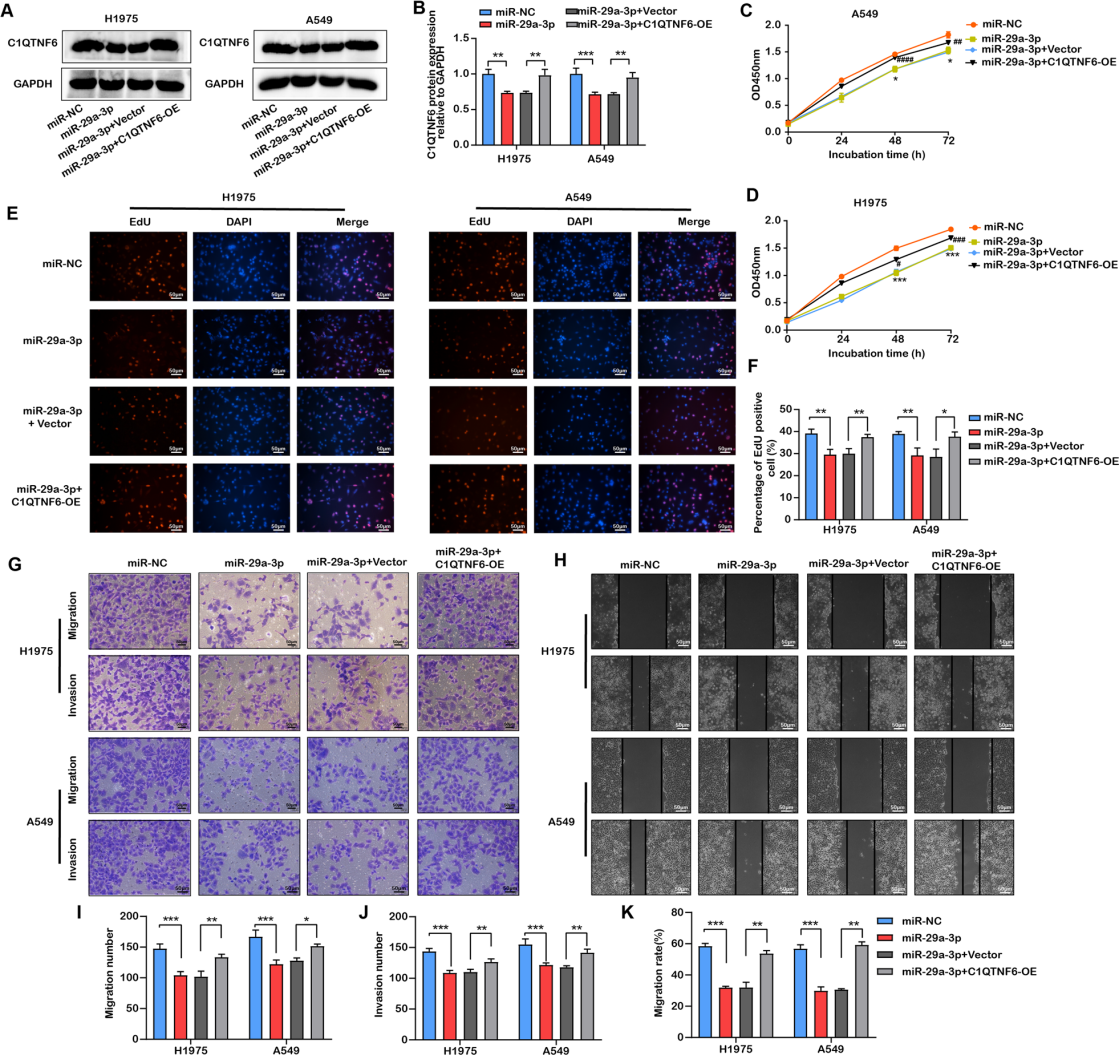
C1QTNF6 upregulation promoted the inhibitory effects of miR‐29a-3p mimics on LUAD cells’ invasive and migrative abilities. **A**, **B** Protein expression levels of C1QTNF6 in H1975 and A549 cells were measured by western blot. **C**–**F** Proliferation of H1975 and A549 cells after transfection with miR‐29a-3p, C1QTNF6-OE and corresponding control were conducted by CCK-8 and EdU assay, scale bar 50 µm. **G**–**K** Migratory and invasive abilities of H1975 and A549 cells after transfection with miR‐29a-3p, C1QTNF6-OE and corresponding control were evaluated by scratch assay and Transwell assay, scale bar 50 µm. OE, overexpression; miR‐29a-3p, miR‐29a-3p mimics; NC, negative control. n = 3. (Data were presented as the mean ± SD of three independent experiments. *p < 0.05, **p < 0.01, ***p < 0.001; ^#^*p* < 0.05, ^**##**^*p *< 0.01, ^**###**^*p *< 0.001, ^**####**^*p *< 0.0001)

To further investigate the function of miR-29a-3p/C1QTNF6 in LUAD cells, gain-of-function and rescue assays were implemented. First, we found that C1QTNF6 protein expression was decreased after miR-29a-3p transfection but was reversed following miR-29a-3p and C1QTNF6 co-transfection (Fig. 5A, B). Further, the results showed that miR-29a-3p overexpression could decrease the cell viability and EdU proliferation positive cell rates, as well as reduce the migratory and invasive rates in LUAD cells when compared with miR-NC groups, which illustrates a tumor suppressor role of upregulated miR-29a-3p. However, the co-upregulation of miR-29a-3p and C1QTNF6 could relieve the suppressive effects of miR-29a-3p on growth, migratory and invasive abilities of LUAD cells (Fig. 5C–K). Together, these data suggested that miR-29a-3p as an upstream regulator could influence biological processes of LUAD cells by regulation of C1QTNF6.

### RNA-Seq investigation of C1QTNF6 inhibition in LUAD cells

**Fig. 6:**
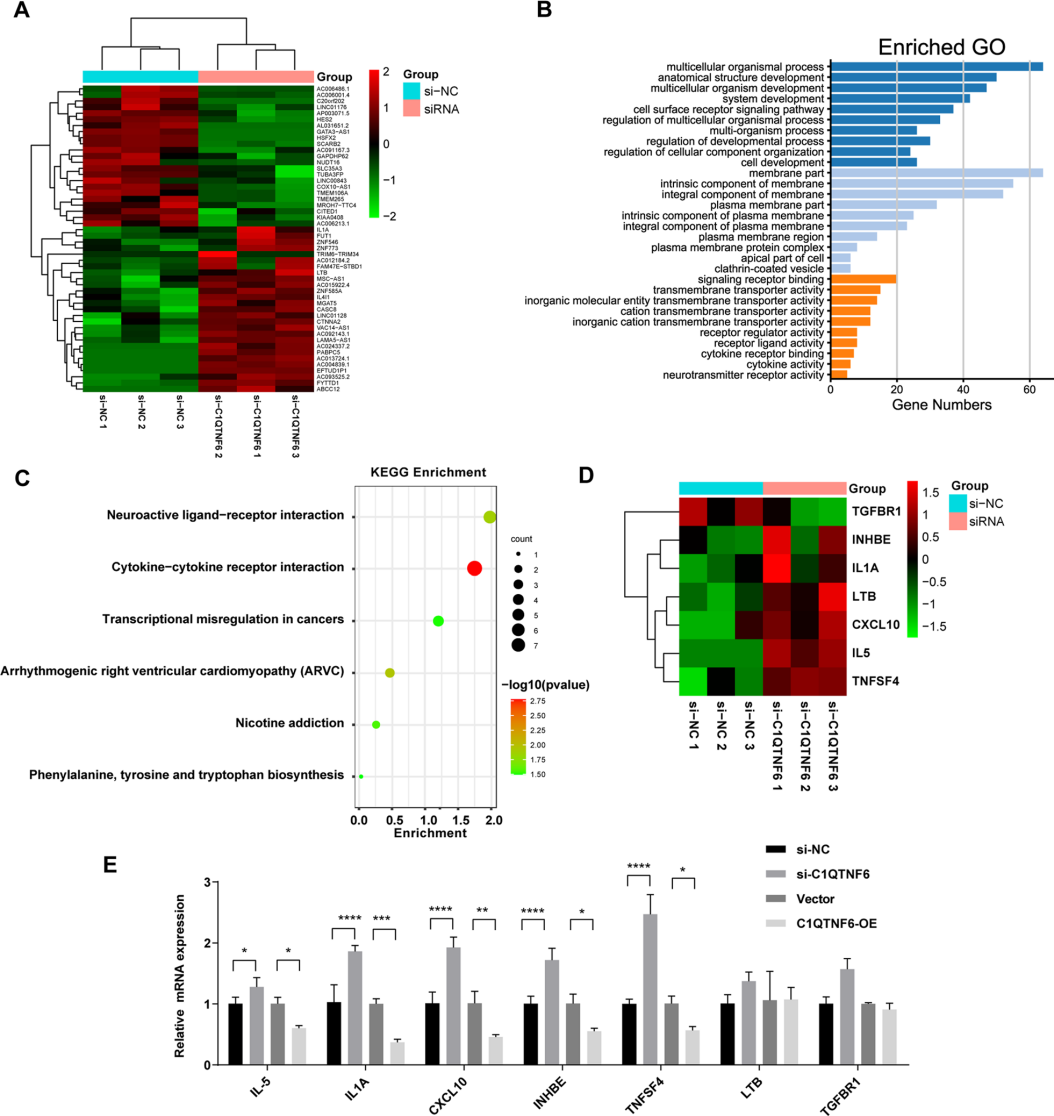
RNA-Seq of C1QTNF6 between A549 knockdown and control cells. **A** Heat map of the differentially expressed genes in the A549 knockdown cells. The red color indicates differentially expressed genes with high expression, while the green color indicates low expression. **B** Gene Ontology analysis of the differentially expressed genes. **C** KEGG pathway analysis on the differentially expressed genes. **D** Heat map of DEGs enriched in the cytokine-cytokine receptor interaction pathway (map04060). **E** mRNA expressions of cytokine-cytokine receptor interaction genes after upregulation or downregulation of C1QTNF6 expression were detected by RT-qPCR. OE, overexpression; miR‐29a-3p, miR‐29a-3p mimics; NC, negative control; 3'UTR, 3'‐untranslated region. n = 3. (Data were presented as the mean ± SD of three independent experiments. *p < 0.05, **p < 0.01, ***p < 0.001, ****p < 0.0001)

To further explore the downstream mechanism of C1QTNF6 in LUAD, RNA-seq analysis was performed in A549 cells with C1QTNF6 knockdown and in control cells. Functional annotation analytics was conducted by gene ontology (GO) enrichment analysis to investigate the biological function of significant differentially expressed genes in the study. Broad classification of GO terms includes three groups: biological process (BP), cellular component (CC), and molecular function (MF). The most significantly enriched processes found out in the study were multicellular organismal process, membrane part, signaling receptor binding and transmembrane transporter activity (Fig. 6A, B). Afterward, Kyoto Encyclopedia of Genes and Genomes (KEGG) pathway analysis was performed to identify the potential signaling pathways. The results indicated that cytokine-cytokine receptor interaction was the most significantly enriched pathway, which mainly involved in 7 genes including LTB, IL-1A, CXCL10, IL-5, TNFSF4, INHBE and TGFBR1 (Fig. 6C). In our sequencing of A549 cells samples, we found that TGFBR1 expression was downregulated, whereas LTB, IL-1A, CXCL10, IL-5, TNFSF4 and INHBE were upregulated in si-C1QTNF6 groups when compared with control groups (Fig. 6D). To further verify whether C1QTNF6 affects cytokine-cytokine receptor interaction pathway, the expression of these 7 genes was detected by RT-qPCR after upregulation or downregulation of C1QTNF6 expression. Our results demonstrated that the mRNA expressions of IL-5, IL-1A, CXCL10, TNFSF4 and INHBE were significantly altered when regulating C1QTNF6 (Fig. 6E), which suggested the essential implications of the cytokine-cytokine receptor interaction pathway in the process of C1QTNF6 regulating tumor progression. Therefore, further studies are required to figure out which cytokines of this signaling pathway are critical in vivo study.

## Discussion

Unconstrained tumor metastasis and dissemination after potential oncogenic mutation entails profound alterations in the essential gene expression of proteins involved in the biological process. Aberrant regulation of C1QTNF6 has been demonstrated to be linked to the carcinogenesis, progression and prognosis in different tumors [17, 35, 36]. Thorough understanding the underlying effect and molecular mechanism of C1QTNF6 in LUAD progression might contribute to developing additional advanced and effective treatment strategies. In the current study, we noticed that C1QTNF6 expression was significantly elevated in LUAD tissues and cell lines and knockdown of C1QTNF6 suppressed the growth and migration in LUAD cell lines. Additionally, miR‐29a-3p, as an upstream regulator of C1QTNF6, could modulate LUAD cells proliferation, migration and invasion abilities by targeting C1QTNF6. Moreover, RNA sequencing analysis of C1QTNF6 knockdown cells revealed C1QTNF6 may be associated with the cytokine-cytokine receptor interaction pathway in LUAD cells.

As a member of C1QTNF family, C1QTNF6 was regarded as a molecular regulator involved in metabolic and inflammatory lesions [10]. C1QTNF6 was up-regulated in humans and mice adipose tissues, overexpression of C1QTNF6 impaired abilities of glucose disposal in response to glucose and insulin resistance in peripheral tissues of wild-type mice. Conversely, C1QTNF6 deletion improved insulin sensitivity and increased metabolism rate and energy expenditure in diet-induced obese mice [37]. Interestingly, C1QTNF6 is now increasingly reported in the field of oncology. For instant, C1QTNF6 was found to be upregulated and to be associated with initiation and progression in multiple malignant tumors including clear cell renal cell carcinoma, gastric cancer, HCC and NSCLC [13, 18, 35, 36]. Similarly, we also observed that C1QTNF6 was highly expressed in stage I LUAD tissues by RNA sequencing as well as by RT-qPCR, western blot and IHC. C1QTNF6 expression was also increased in LUAD cell lines, especially in H1975 and A549 cell lines. Then we established the H1975 and A549 C1QTNF6 downregulation cell lines by RNA interference. The results showed that C1QTNF6 knockdown significantly suppressed cell growth and the migratory and invasive abilities in LUAD cells, which indicated an important function of C1QTNF6 in progression of LUAD. However, the specific underlying mechanism and pathways of C1QTNF6 in LUAD have not been reported. Therefore, we further explored the molecular mechanism and its upstream and downstream factors.

In recent years, miRNAs have identified as vital regulators involved in most of cellular processes from cell metabolism, proliferation and differentiation to cell apoptosis and death [38–40]. Deregulated miRNA expression leads to tumor dissemination and progression by promoting acquisition of cancer hallmark traits [41]. MiR-29a-3p, one member of miR-29 family, has drawn wide attention as a promising tumor suppressor in several kinds of cancers. Previous study pointed out that the expression of miR-29a-3p was markedly lower in prostate cancer (PCa) tissues than adjacent non-tumorous tissues and miR-29a-3p overexpression could inhibit the proliferation, migration and invasion of PCa cells [42]. Moreover, miR-29a-3p mediated its tumor-suppressive activity and suppressed the tumorigenicity in HeLa cells by directly targeting smad nuclear interacting protein 1 (SNIP1) [43]. However, there was no previous report investigated expression levels and regulatory roles of miR-29a-3p in LUAD tissues. In the present study, we predicted that miR-29a-3p may be a potential upstream of C1QTNF6 and proved that miR-29a-3p was significantly downregulated in stage I LUAD tissues. The dual-luciferase reporter assay also demonstrated that miR-29a-3p bound to the 3'UTR of C1QTNF6, indicating that C1QTNF6 was a target gene of miR-29a-3p. Therefore, we further explored the function of miR-29a-3p in LUAD cells. The results showed that miR-29a-3p overexpression could also inhibit cell proliferation, migration and invasion in LUAD cells.

MiRNAs have been ubiquitously regarded as upstream regulators of mRNAs, which could repress mRNAs post-transcriptionally through binding to the 3'UTR of the mRNAs and affect the translational activity and biological functions of target genes [44]. Previous reports have indicated miRNAs may participate in malignant phenotype progression and predict as diagnostic and prognostic biomarkers via degrading mRNA targets in lung cancer [45, 46]. To verify whether miR-29a-3p acts as an upstream molecule of C1QTNF6 to modulate biological processes of LUAD cells, rescue assays were performed. The results indicated that co-transfection of miR-29a-3p and C1QTNF6 could alleviate inhibitory activities of miR-29a-3p on proliferation, migratory and invasive abilities of LUAD cells, suggesting that miR‐29a-3p may serve as a direct binding partner by targeting C1QTNF6.

Another interesting finding was that decreased C1QTNF6 expression was mainly associated with cytokine-cytokine receptor interaction pathway in LUAD cell lines by RNA sequencing. Cytokines are a group of bioactive proteins with small molecular weight (5–70 kDa) and predominantly secreted from a range of cells including macrophages, lymphocytes, natural killer (NK) cells, mast cells and various stromal cells [47]. They act as important mediators in dynamic regulation of immune system and play distinguished roles in initiating and promoting tumor progression [48, 49]. In the present study, seven DEGs identified between C1QTNF6 knockdown cells and control cells were enriched in cytokine-cytokine receptor interaction. Further RT-qPCR analysis also demonstrated that IL-5, IL-1A, CXCL10, TNFSF4 and INHBE were significantly changed in LUAD cells after C1QTNF6 knockdown or overexpression, which suggested the importance of the cytokine-cytokine receptor interaction pathway in the process of C1QTNF6 regulating tumor progression. Similarly, He et al. reported that CXCR4 and CCR2 were enriched in the cytokine-cytokine receptor interaction pathway, which could be regulated by miR-122 and involved in the development of HCC ^50^. Therefore, the results of our study may provide a theoretical and empirical principle for targeted reactivation of C1QTNF6 in the development of LUAD.

Despite we discovered C1QTNF6 exerted impact on initiation and progression of LUAD regulated by miR-29a-3p and closely associated with cytokine-cytokine receptor interaction pathway, we need to acknowledge that there are still some limitations. First, Xenograft tumor construction was not performed in the present study. Second, we did not perform prognostic analysis as well as correlation between C1QTNF6 and clinical characteristics due to insufficient sample size of pathological tissues. Third, for the complex constituent of cytokine-cytokine receptor interaction pathway, the regulatory relationships and specific mechanisms between the pathway and C1QTNF6 in LUAD has not been revealed. Additional *in vivo* verification and clinical research is required, and the significance of cytokine-cytokine receptor interaction pathway in LUAD is thoroughly to be explored.

## Conclusion

In summary, our study first confirmed that downregulation of C1QTNF6 could inhibit tumorigenesis and progression in early-stage LUAD cells negatively regulated by miR‐29a-3p. These findings identified that C1QTNF6 and miR‐29a-3p exerted impact on initiation and progression of early-stage LUAD, which could enhance intimate understanding of the underlying mechanisms and may provide a novel and promising therapeutic target for early-stage LUAD.

## Supplementary Information

**Additional file 1. Table S1**. List of primer sequences and** Figure S1**.

## Data Availability

The datasets generated during the current study are not publicly available due to concerns regarding patient confidentiality and proprietary information but are available upon reasonable request from the corresponding author. we provided a reviewer link of unpublished BioProject. Use the following URL: https://dataview.ncbi.nlm.nih.gov/object/PRJNA732584?reviewer=jo5ph00rjrkr7u19thhft8eho.

## Abbreviations

C1QTNFs: C1q/tumor necrosis factor-related proteins
LUAD: lung adenocarcinoma
NSCLC: Non-small cell lung cancer
HCC: Hepatocellular carcinoma
BC: Bladder cancer
miRNAs: MicroRNAs
mRNA: Messenger RNAs
FBS: Fetal bovine serum
IHC: Immunohistochemistry
CCK-8: Cell counting kit-8
PI: Propidium iodide
RT-qPCR: Quantitative reverse transcription polymerase chain reaction
GO: Gene ontology
KEGG: Kyoto encyclopedia of genes and genomes
TCGA: The cancer genome atlas
DEGs: Differentially expressed genes
BP: Biological process
CC: Cellular component
MF: Molecular function
WT: Wild type
Mut: Mutant

## References

[1] Arbour KC, Riely GJ (2019). Systemic therapy for locally advanced and metastatic non-small cell lung cancer: a review. JAMA.

[2] Leiter U, Keim U, Garbe C (2019). Cancer statistics, 2019. CA Cancer J Clin.

[3] Saynak M, Veeramachaneni NK, Hubbs JL, Nam J, Qaqish BF, Bailey JE, Chung W (2011). Local failure after complete resection of N0–1 non-small cell lung cancer. Lung Cancer (Amsterdam, Netherlands).

[4] Taylor MD, Nagji AS, Bhamidipati CM, Theodosakis N, Kozower BD, Lau CL, Jones DR (2012). Tumor recurrence after complete resection for non-small cell lung cancer. Ann Thorac Surg.

[5] Liu L, Zhang L, Wang J, Zhao X, Xu Q, Lu Y, Zuo Y, Chen L, Du J, Lian Y, Zhang Q (2018). Downregulation of TRIM28 inhibits growth and increases apoptosis of nude mice with non-small cell lung cancer xenografts. Mol Med Rep.

[6] Jemal A, Bray F, Center MM, Ferlay J, Ward E (2011). Global cancer statistics. CA Cancer J Clin.

[7] Peterson JM, Wei Z, Wong GW (2010). C1q/TNF-related protein-3 (CTRP3), a novel adipokine that regulates hepatic glucose output. J Biol Chem.

[8] Li Q, Wang L, Tan W, Peng Z, Luo Y, Zhang Y, Zhang G, Na D, Jin P, Shi T, Ma D (2011). Identification of C1qTNF-related protein 4 as a potential cytokine that stimulates the STAT3 and NF-κB pathways and promotes cell survival in human cancer cells. Cancer Lett.

[9] Wu W, Ji M, Xu K, Zhang D, Yin Y, Huang X, Peng Y, Zhang J (2020). Knockdown of CTRP6 reduces the deposition of intramuscular and subcutaneous fat in pigs via different signaling pathways. Biochim Biophysica Acta Mol Cell Biol Lipids.

[10] Schäffler A, Buechler C (2012). CTRP family: linking immunity to metabolism. Trends Endocrinol Metab TEM.

[11] Han M, Wang B, Zhu M, Zhang Y (2019). C1QTNF6 as a novel biomarker regulates cellular behaviors in A549 cells and exacerbates the outcome of lung adenocarcinoma patients: in vitro cellular & developmental biology. Animal.

[12] Wong GW, Krawczyk SA, Kitidis-Mitrokostas C, Revett T, Gimeno R, Lodish HF (2008). Molecular, biochemical and functional characterizations of C1q/TNF family members: adipose-tissue-selective expression patterns, regulation by PPAR-gamma agonist, cysteine-mediated oligomerizations, combinatorial associations and metabolic functions. Biochem J.

[13] Wan X, Zheng C, Dong L (2019). Inhibition of CTRP6 prevented survival and migration in hepatocellular carcinoma through inactivating the AKT signaling pathway. J Cell Biochem.

[14] Ghai R, Waters P, Roumenina LT, Gadjeva M, Kojouharova MS, Reid KB, Sim RB, Kishore U (2007). C1q and its growing family. Immunobiology.

[15] Murayama MA, Kakuta S, Maruhashi T, Shimizu K, Seno A, Kubo S, Sato N, Saijo S, Hattori M, Iwakura Y (2014). CTRP3 plays an important role in the development of collagen-induced arthritis in mice. Biochem Biophys Res Commun.

[16] Chi L, Hu X, Zhang W, Bai T, Zhang L, Zeng H, Guo R, Zhang Y, Tian H (2017). Adipokine CTRP6 improves PPARγ activation to alleviate angiotensin II-induced hypertension and vascular endothelial dysfunction in spontaneously hypertensive rats. Biochem Bioph Res Commun.

[17] Zhu X, Tong H, Gao S, Yin H, Zhu G, Li X, He W, Gou X (2020). C1QTNF6 overexpression acts as a predictor of poor prognosis in bladder cancer patients. Biomed Res Int.

[18] Zhang W, Feng G (2021) C1QTNF6 regulates cell proliferation and apoptosis of NSCLC in vitro and in vivo. Biosci Rep 41(1) 10.1042/BSR20201541 PMC7805025 33269376

[19] Bartel DP (2004). MicroRNAs: genomics, biogenesis, mechanism, and function. Cell.

[20] Shenoy A, Blelloch RH (2014). Regulation of microRNA function in somatic stem cell proliferation and differentiation. Nat Rev Mol Cell Biol.

[21] Lg L, Ch C, La C (1986). Thiazole orange: a new dye for reticulocyte analysis. Cytometry.

[22] Lynam-Lennon N, Maher SG, Reynolds JV (2009). The roles of microRNA in cancer and apoptosis. Biol Rev Camb Philos Soc.

[23] Cp B, Hs S, Gj G (2016). A network-biology perspective of microRNA function and dysfunction in cancer. Nat Rev Genet.

[24] Volinia S, Calin GA, Liu CG, Ambs S, Cimmino A, Petrocca F, Visone R, Iorio M, Roldo C, Ferracin M, Prueitt R (2006). A microRNA expression signature of human solid tumors defines cancer gene targets. Proc Natl Acad Sci USA.

[25] Xie Y, Wang Y, Li J, Hang Y, Jaramillo L, Wehrkamp CJ, Phillippi MA, Mohr AM, Chen Y, Talmon GA (2018). Cholangiocarcinoma therapy with nanoparticles that combine downregulation of MicroRNA-210 with inhibition of cancer cell invasiveness. Theranostics.

[26] Zhou JS, Yang ZS, Cheng SY, Yu JH, Huang CJ, Feng Q (2020). miRNA-425-5p enhances lung cancer growth via the PTEN/PI3K/AKT signaling axis. BMC Pulm Med.

[27] Han S, Wang Z, Liu J, Wang HM (2021). miR-29a-3p-dependent COL3A1 and COL5A1 expression reduction assists sulforaphane to inhibit gastric cancer progression. Biochem Pharmacol.

[28] Zhang H, Wang Y, Ding H (2021). COL4A1, negatively regulated by XPD and miR-29a-3p, promotes cell proliferation, migration, invasion and epithelial-mesenchymal transition in liver cancer cells. Clin Transl Oncol Off Publ Fed Spanish Oncol Soc Natl Cancer Inst Mexico.

[29] Kong Z, Wan X, Lu Y, Zhang Y, Huang Y, Xu Y, Liu Y, Zhao P, Xiang X, Li L, Li Y (2020). Circular RNA circFOXO3 promotes prostate cancer progression through sponging miR-29a-3p. J Cell Mol Med.

[30] Livak KJ, Schmittgen TD (2001). Analysis of relative gene expression data using real-time quantitative PCR and the 2(-delta delta C(T)) method. Methods (San Diego, CA).

[31] Young AR, Narita M, Ferreira M, Kirschner K, Sadaie M, Darot JF, Tavaré S, Arakawa S, Shimizu S, Watt FM, Narita M (2009). Autophagy mediates the mitotic senescence transition. Gene Dev.

[32] Liu Y, Chen X, Cheng R, Yang F, Yu M, Wang C, Cui S, Hong Y, Liang H, Liu M, Zhao C (2018). The Jun/miR-22/HuR regulatory axis contributes to tumourigenesis in colorectal cancer. Mol Cancer.

[33] Du Z, Zhou X, Ling Y, Zhang Z, Su Z (2010). agriGO: a GO analysis toolkit for the agricultural community. Nucleic Acids Res.

[34] Kanehisa M, Furumichi M, Tanabe M, Sato Y, Morishima K (2017). KEGG: new perspectives on genomes, pathways, diseases and drugs. Nucleic Acids Res.

[35] Lin W, Chen X, Chen T, Liu J, Ye Y, Chen L, Qiu X, Chia-Hsien Cheng J, Zhang L, Wu J, Qiu S (2020). C1QTNF6 as a novel diagnostic and prognostic biomarker for clear cell renal cell carcinoma. DNA Cell Biol.

[36] Qu HX, Cui L, Meng XY, Wang ZJ, Cui YX, Yu YP, Wang D, Jiang XJ (2019). C1QTNF6 is overexpressed in gastric carcinoma and contributes to the proliferation and migration of gastric carcinoma cells. Int J Mol Med.

[37] Lei X, Seldin MM, Little HC, Choy N, Klonisch T, Wong GW (2017). C1q/TNF-related protein 6 (CTRP6) links obesity to adipose tissue inflammation and insulin resistance. J Biol Chem.

[38] He L, He X, Lowe SW (2007). microRNAs join the p53 network–another piece in the tumour-suppression puzzle. Nat Rev Cancer.

[39] Riesco-Eizaguirre G, Wert-Lamas L, Perales-Paton J, Sastre-Perona A, Fernandez LP, Santisteban P (2015). The miR-146b-3p/PAX8/NIS regulatory circuit modulates the differentiation phenotype and function of thyroid cells during carcinogenesis. Cancer Res.

[40] Wang X, He Y, Mackowiak B, Gao B (2021). MicroRNAs as regulators, biomarkers and therapeutic targets in liver diseases. Gut.

[41] Reda El Sayed S, Cristante J, Guyon L, Denis J, Chabre O, Cherradi N (2021). MicroRNA therapeutics in cancer current advances and challenges. Cancers.

[42] Liao B, Chen S, Li Y, Yang Z, Yang Y, Deng X, Ke S (2021). LncRNA BLACAT1 promotes proliferation, migration and invasion of prostate cancer cells via regulating miR-29a-3p/DVL3 Axis. Technol Cancer Res Treat.

[43] Chen Y, Zhang W, Yan L, Zheng P, Li J (2020). miR-29a-3p directly targets Smad nuclear interacting protein 1 and inhibits the migration and proliferation of cervical cancer HeLa cells. PEERJ.

[44] Je R, Jj W (2017). Nuclear microRNAs in normal hemopoiesis and cancer. J Hematol Oncol.

[45] Tang S, Li S, Liu T, He Y, Hu H, Zhu Y, Tang S (2021). MicroRNAs: emerging oncogenic and tumor-suppressive regulators, biomarkers and therapeutic targets in lung cancer. Cancer Lett.

[46] Rezaei S, Mahjoubin-Tehran M, Aghaee-Bakhtiari SH, Jalili A, Movahedpour A, Khan H, Moghoofei M, Shojaei Z, Hamblin MR, Mirzaei H (2020). Autophagy-related MicroRNAs in chronic lung diseases and lung cancer. Critical Rev Oncol/Hematol.

[47] Jj O, Pj M (2008). Cytokine signaling modules in inflammatory responses. Immunity.

[48] Burska A, Boissinot M, Ponchel F (2014). Cytokines as biomarkers in rheumatoid arthritis. Mediat Inflamm.

[49] Tong Y, Song Y, Deng S (2019). Combined analysis and validation for DNA methylation and gene expression profiles associated with prostate cancer. Cancer Cell Int.

[50] He B, He Y, Shi W, Gong S, Chen X, Xiao J, Gu J, Ding W, Wang Y (2017). Bioinformatics analysis of gene expression alterations in microRNA-122 knockout mice with hepatocellular carcinoma. Mol Med Rep.

