# Supplementary material for: C1QTNF6 regulated by miR‐29a-3p promotes proliferation and migration in stage I lung adenocarcinoma
Source: BMC Pulm Med. 2022 Jul 25;22:285. doi: 10.1186/s12890-022-02055-2 (PMC9310408; doi:10.1186/s12890-022-02055-2)
Supplement: Supplementary file 1 — Additional file 1. Table S1. List of primer sequences and Figure S1. [file 12890_2022_2055_MOESM1_ESM.docx]

***Supplementary Material***

| Gene | Forward (5’-3’) | Reverse (5’-3’) |
| --- | --- | --- |
| C1QTNF6 | TGCCTGAGATCAGACCCTACA | GCCCACTGAGAAGGCGAAG |
| Si-NC | UUCUCCGAACGUGUCACGUdTdT | ACGUGACACGUUCGGAGAAdTdT |
| Si-C1QTNF6-1 | CAACGACUUCGACACCUACdTdT | GUAGGUGUCGAAGUCGUUGdTdT |
| Si-C1QTNF6-2 | GAAAGGGUCUUUGUGAACCdTdT | GGUUCACAAAGACCCUUUCdTdT |
| Si-C1QTNF6-3 | GAUGUGUGAGAUCCCUAUGdTdT | CAUAGGGAUCUCACACAUCdTdT |
| Stem-loop primer of miR-29a-3p | GTCGTATCCAGTGCGTGTCGTGGAGTCGGCAATTGCACTGGATACGACAACACTGA | |
| MiR-29a-3p | GGGCTAGCACCATTTGAAATC | CCAGTGCGTGTCGTGGAGT |
| MiR-29a-3p mimics NC | UCACAACCUCCUAGAAAGAGUAGA | UCUACUCUUUCUAGGAGGUUGUGA |
| MiR-29a-3p mimics | UAGCACCAUCUGAAAUCGGUUA | UAACCGAUUUCAGAUGGUGCUA |
| MiR-29a-3p inhibitor NC |  | UCUACUCUUUCUAGGAGGUUGUGA |
| MiR-29a-3p inhibitor |  | UAACCGAUUUCAGAUGGUGCUA |
| U6 | CTCGCTTCGGCAGCACA | AACGCTTCACGAATTTGCGT |
| GAPDH | CTCCTGCACCACCAACTGCTTAG | GACGCCTGCTTCACCACCTTC |
| IL-5 | TTGACTCTCCAGTGTGCCTATTCCCTGAA | AAATCACCAACTGTGCACTGAA |
| IL-1A | CAAACTGATGAAGCTCGTCA | TCTCCTTGAGCGCTCACGAA |
| CXCL10 | AACTGTACGCTGTACCTGCAT | GCATCGATTTTGCTCCCCTC |
| INHBE | ACTACAGCCAGGGAGTGTGG | AGTGAGCAGGGAGCTGTAGG |
| TNFSF4 | ATCTCCCTGAAGGGCTACTT | GTTGACAGACCTGACCTTCTTC |
| LTB | AGGAGCCACTTCTCTGGTGA | CTCTGGCAGCTTCTGAAACC |
| TGFBR1 | GGTTCCGTGAGGCAGAGATTTA | CTGACACCAACCAGAGCTGAGT |

**Table S1.** Primer sequence information


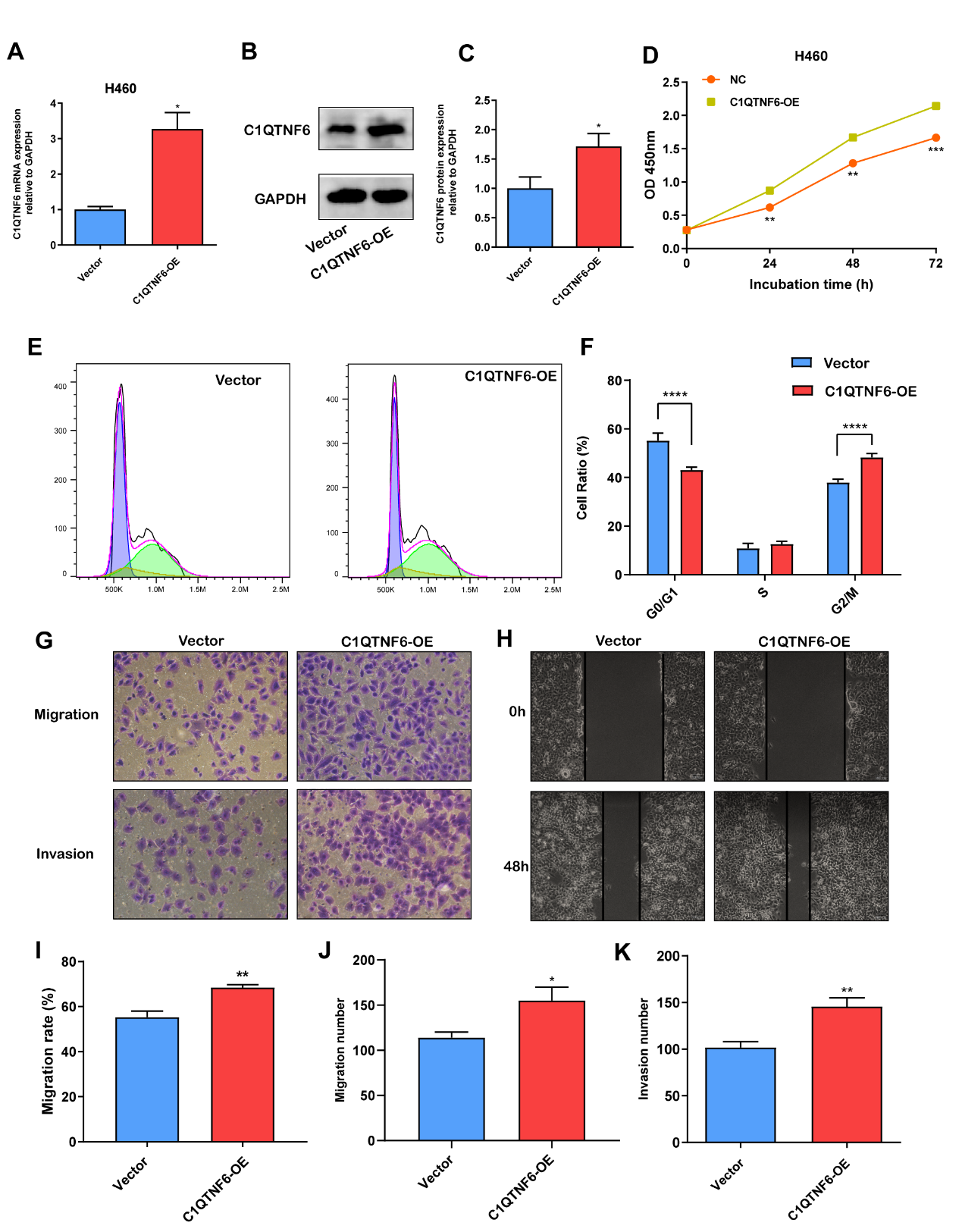


**Figure S1.** C1QTNF6 overexpression promoted cell growth and the migratory and invasive abilities in LUAD cells. (A-C) Overexpression efficiency of C1QTNF6 were assessed by RT-qPCR and western blot. (D) Growth curves for C1QTNF6-OE group and control group were performed by CCK-8. (E-F) The cell cycles of H460 cells upon transfection with C1QTNF6-OE and empty vector were analyzed using flow cytometry. (G-K) Migration and invasion of H460 cells were determined by scratch experiments and Transwell, scale bar 50 µm. OE, overexpression. n = 3. (Data are presented as the mean ± SD of three independent experiments. **p* < 0.05, ***p* < 0.01, ****p* < 0.001, *****p* < 0.0001).
